# Alkylated albumin-derived dipeptide C(-HETE)P derivatized by propionic anhydride as a biomarker for the verification of poisoning with sulfur mustard

**DOI:** 10.1007/s00216-021-03454-w

**Published:** 2021-07-02

**Authors:** Annika Richter, Markus Siegert, Horst Thiermann, Harald John

**Affiliations:** 1 Department of Chemistry, Humboldt-Universität zu Berlin, Brook-Taylor-Straße 2, 12489 Berlin, Germany; 2 Bundeswehr Institute of Pharmacology and Toxicology, Neuherbergstrasse 11, 80937 Munich, Germany

**Keywords:** Biomarker, Chemical warfare agent, HETE moiety, Protein adduct, Propionic anhydride, Verification

## Abstract

Sulfur mustard (SM) is a banned chemical warfare agent recently used in the Syrian Arab Republic conflict causing erythema and blisters characterized by complicated and delayed wound healing. For medical and legal reasons, the proof of exposure to SM is of high toxicological and forensic relevance. SM reacts with endogenous human serum albumin (HSA adducts) alkylating the thiol group of the cysteine residue C^34^, thus causing the addition of the hydroxyethylthioethyl (HETE) moiety. Following proteolysis with pronase, the biomarker dipeptide C(-HETE)P is produced. To expand the possibilities for verification of exposure, we herein introduce a novel biomarker produced from that alkylated dipeptide by derivatization with propionic anhydride inducing the selective propionylation of the N-terminus yielding PA-C(-HETE)P. Quantitative derivatization is carried out at room temperature in aqueous buffer within 10 s. The biomarker was found to be stable in the autosampler at 15 °C for at least 24 h, thus documenting its suitability even for larger sets of samples. Selective and sensitive detection is done by micro liquid chromatography-electrospray ionization tandem-mass spectrometry (μLC-ESI MS/MS) operating in the selected reaction monitoring (SRM) mode detecting product ions of the single protonated PA-C(-HETE)P (m/z 379.1) at m/z 116.1, m/z 137.0, and m/z 105.0. The lower limit of detection corresponds to 32 nM SM in plasma in vitro and the limit of identification to 160 nM. The applicability to real exposure scenarios was proven by analyzing samples from the Middle East confirming poisoning with SM.

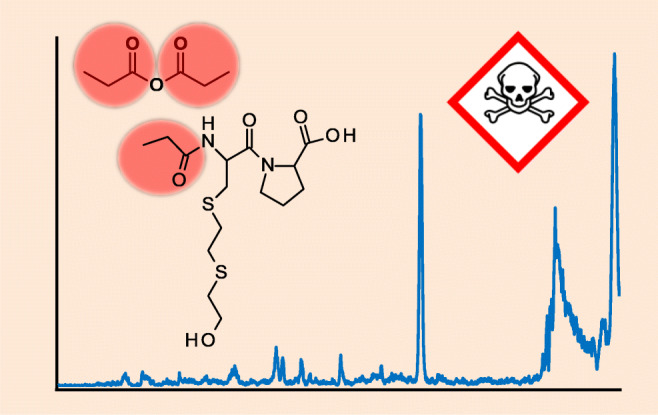

## Introduction

Sulfur mustard (SM, bis(2-chloroethyl)-sulfide) is a chemical warfare agent (CWA) and belongs to the class of vesicants [1, 2]. It has been used for more than 100 years with its first usage in 1917 during World War I in Ypres (Belgium) and most recently during the Syrian Arab Republic conflict [3–5]. Skin areas exposed to SM may develop erythema and painful blisters that are characterized by complicated and delayed wound healing [6]. The use, stockpiling, and production of SM is prohibited by the Chemical Weapons Convention (CWC) implemented by the Organisation for the Prohibition of Chemical Weapons (OPCW) [7]. Nevertheless, exposure to SM still represents a threat to the military as well as to the civilian population [8, 9]. Therefore, reliable, rapid, simple, selective, and sensitive bioanalytical methods are demanded for the verification of SM exposure and to provide evidence for the violation of the CWC [7].

In vivo, SM can either be hydrolyzed to the non-toxic thiodiglycol (TDG) (detectable in urine for up to 1 week after exposure) or it may alkylate nucleophiles in, e.g., DNA and proteins like keratin, hemoglobin, or human serum albumin (HSA) [10–13]. These protein adducts represent long-lived analytical targets of SM exposure (half-life of HSA adduct at Cys^34^: 22 days) [14, 15]. All adducts are characterized by the presence of the SM-derived hydroxyethylthioethyl (HETE) moiety attached to nucleophilic sites of the protein [12, 14]. Enzymatic cleavage of the HETE-HSA adduct with proteinase K generates the alkylated tripeptide C(-HETE)PF [14] and cleavage with pronase produces the alkylated dipeptide C(-HETE)P [16–18] that may be monitored by micro liquid chromatography-electrospray ionization tandem-mass spectrometry (μLC-ESI MS/MS). These peptide biomarkers have been successfully used in interlaboratory exercises of the OPCW [18] as well as to investigate real case plasma samples drawn in the Middle East in 2015 [4].

In principle, the extension of the toolbox for verification methods is a core requirement to optimize established methods or include alternative analytical targets. Accordingly, in 2019, Chen et al. introduced a method to derivatize the C(-HETE)P biomarker with benzyl chloroformate (BCF) [19]. This procedure prolongs the time for sample preparation significantly (2 h of derivatization at 4 °C) and makes use of the toxic and environmentally harmful BCF. In addition, the derivatization yield was reported to be as low as 47%, thus reducing the detectable amount of the biomarker [19]. Furthermore, no application to real cases of SM poisoning has been reported so far. Avoiding these restrictions, we herein present a novel, rapid, and quantitative method to propionylate C(-HETE)P at its N-terminus with the use of propionic anhydride and subsequent μLC-ESI MS/MS analysis. The biomarker was characterized, compared to already established biomarkers and successfully applied to plasma samples from real cases of SM intoxication [4].

## Materials and methods

### Chemicals and reagents

Acetone (p.A.), acetonitrile (ACN, LC-MS grade), dioxane (99.8%), isopropanol (iPrOH, LC-MS grade), and water (LiChrosolv) were purchased from Merck (Darmstadt, Germany). Formic acid (FA, ≥ 98%) and NaOCl solution for decontamination (12% Cl_2_) were from Carl Roth (Karlsruhe, Germany). Propionic anhydride (≥ 99%), trifluoroacetic acid (TFA, ≥ 99%), triethylamine (TEA, ≥ 99.5%) and phosphate-buffered saline (PBS) were from Sigma-Aldrich (Steinheim, Germany), pronase from *Streptomyces griseus* from Roche (lot no. 70327222, Mannheim, Germany), NH_4_HCO_3_ (ultra-grade, ≥ 99.5%) from Fluka (Buchs, Switzerland), and three-fold deuterated atropine (d_3_-Atr) from CDN Isotopes (Pointe Claire, Quebec, Canada). The d_3_-Atr working solution (3 ng/mL) was prepared in 0.5% v/v FA. Human ethylenediaminetetraacetic acid (EDTA) plasma from different individuals was purchased from in.vent Diagnostica (Hennigsdorf, Germany). SM was made available by the German Ministry of Defense and tested for integrity and purity in-house by nuclear magnetic resonance (NMR) spectroscopy.

### Incubation of plasma with SM

EDTA-plasma (980 μL) was mixed with SM solution (20 μL in iPrOH) and incubated for 2 h at 37 °C under gentle shaking. SM concentrations in plasma of 50 μM and 12.5 μM were used to produce references. Additional concentrations of SM were used as described below (section: linear range, limit of detection, and limit of identification). Blanks were incubated with 20 μL iPrOH only. Samples were stored if necessary at − 20 °C until further processing.

### Precipitation of proteins from EDTA-plasma samples

EDTA-plasma (50 μL) was mixed with acetone (250 μL) in a 1.5 mL reaction vial, vortex mixed for 10 s and centrifuged afterwards (3500*g*, 3 min at room temperature, RT). The supernatant was removed and the protein pellet washed with acetone (250 μL) by sonication for 2 min. After centrifugation (3500*g*, 3 min, RT) and removal of the supernatant, the pellet was air-dried prior to proteolysis.

### Proteolysis of the albumin adduct with pronase

The protein pellet obtained from EDTA-plasma was suspended in 50 mM NH_4_HCO_3_ (200 μL). Pronase solution (50 μL, 10 mg/mL in 50 mM NH_4_HCO_3_) was added prior to incubation for 2 h at 50 °C. Afterwards, ACN (750 μL) was added followed by centrifugation (13,000*g*, 5 min, RT). The supernatant was removed, dried by vacuum, and redissolved in 50 mM NH_4_HCO_3_ (250 μL). An aliquot (50 μL) was mixed with 10 μL water and diluted 1:3 with d_3_-Atr working solution as an internal standard (IS) for subsequent μLC-ESI MS/MS analysis in the selected reaction monitoring (SRM) mode (sample volume 20 μL). Another 50 μL aliquot was subjected to derivatization.

### Standard protocol for the derivatization of C(-HETE)P with propionic anhydride

A portion (50 μL) of the redissolved dried supernatant after proteolysis containing C(-HETE)P was mixed with propionic anhydride (10 μL, RT) in a 0.5-mL reaction vial and vortex mixed for 10 s. An aliquot (30 μL) of the analyte solution was diluted 1:3 with d_3_-Atr working solution for subsequent analysis of 20 μL by μLC-ESI high-resolution tandem-mass spectrometry (MS/HRMS) in the product ion scan (PIS) mode and by μLC-ESI MS/MS in PIS and SRM mode.

### Optimization of the derivatization conditions

The derivatization reaction was optimized with respect to the amount of propionic anhydride, reaction time, and water amount to stop the reaction (quenching). A portion (50 μL) of the redissolved dried supernatant after proteolysis of reference (50 μM SM) plasma proteins containing C(-HETE)P was used for each experiment. Peak areas obtained from the extracted ion chromatogram (XIC) of the qualifying ion at m/z 116.1 (Qual 1) of PA-C(-HETE)P were determined. The amount of propionic anhydride was varied using 3 μL, 5 μL, 10 μL, 25 μL, and 50 μL. The reaction time was varied and stopped by quenching the reaction mixture with 125 μL water after 10 s, 30 s, 1 min, 2 min, 3 min, and 5 min of derivatization.

### Derivatization of C(-HETE)P with BCF

The derivatization was carried out according to Chen et al. [19]. An aliquot (50 μL) of the redissolved dried supernatant after proteolysis of reference (50 μM SM) plasma proteins was transferred into a 0.5-mL reaction vial, cooled to 4 °C for 5 min, and subsequently mixed with BCF (5 μL, 200 mg/mL in dioxane, 4 °C). The solution was incubated for 30 min at 4 °C under gentle shaking. The derivatization was terminated by the addition of 2 μL TFA (20% v/v) and 3 μL H_2_O. An aliquot (30 μL) was diluted 1:3 with d_3_-Atr working solution for subsequent μLC-ESI MS/MS (SRM) analysis.

### μLC-ESI MS/HRMS (PIS) analysis

Analysis by MS/HRMS was carried out to determine the accurate masses of product ions using the PIS mode. For chromatographic separation, a micro LC 200 system (Eksigent Technologies, Dublin, CA, USA) equipped with a HTC-xt DLW autosampler (CTC Analytics, Zwingen, Switzerland) and a 20-μL sample loop (Sunchrom, Friedrichsdorf, Germany) were used. Peptides were separated at 60 °C on an Acquity UPLC HSS T3 column (50 × 1.0 mm I.D., 1.8 μm, 100 Å; Waters, Eschborn, Germany) protected by a precolumn (Security Guard Ultra cartridge C18 peptide; Phenomenex, Aschaffenburg, Germany) applying a binary gradient mobile phase with 30 μL/min of solvent A (0.05% v/v FA) and solvent B (ACN/H_2_O 80:20 v/v, 0.05% v/v FA): t [min]/B [%]: 0/2, 11/30, 11.5/95, 13.5/95, 14/2, 15/2.

The μLC system was coupled via an ESI interface (Turbo V source, + 5.5 kV) to a high-resolution hybrid mass spectrometer (TT5600^+^, ABSciex, Darmstadt, Germany) that combines a quadrupole for precursor ion selection with a time-of-fight (TOF) mass analyzer for product ion distinction. The mass spectrometer operated in the positive PIS mode after collision-induced dissociation (CID) with high sensitivity in the range from m/z 50 to m/z 700 for C(-HETE)P, propionylated-C(-HETE)P, herein referred to as PA-C(-HETE)P, and the IS. The following MS settings were applied: declustering potential (DP) 60 V, curtain gas (CUR) 30 psi (2.07 × 10^5^ Pa), temperature 300 °C, source gas 1 (GS1) 50 psi (3.45 × 10^5^ Pa), source gas 2 (GS2) 50 psi (3.45 × 10^5^ Pa), and collision energy (CE) 30 V. The mass spectrometer was also connected with a calibrant delivery system (CDS, ABSciex) via an atmospheric pressure chemical ionization ion source inlet to infuse a calibrant solution (“APCI positive calibration solution,” ABSciex) after each fifth μLC run with a flow of 500 μL/min. The entire μLC-ESI MS/HRMS system was controlled by the Analyst TF 1.7.1 software (ABSciex) and the Eksigent control software (version 4.2).

### μLC-ESI MS/MS analysis in PIS and SRM mode

The presented procedure is based on the technique described by Blum et al. [20]. In brief, chromatography was carried out using a M5 microLC system comprising an integrated autosampler (15 °C) allowing 20 μL sample volume injection (ABSciex), applying the same conditions of mobile and stationary phase as described above for μLC-ESI MS/HRMS analysis. Using an ESI interface working in positive mode (OptiFlow turbo V source, + 5 kV) the QTrap 6500^+^ mass spectrometer (ABSciex) was coupled to monitor product ions after CID of analytes using nitrogen as collision gas. The following MS settings were applied: DP 60 V, CUR 30 psi (2.07 × 10^5^ Pa), temperature 200 °C, GS1 50 psi (3.45 × 10^5^ Pa), GS2 60 psi (4.14 × 10^5^ Pa), entrance potential (EP) 10 V, cell exit potential (CXP) 10 V, and dwell time 50 ms. The single protonated precursor ions of the biomarkers were fragmented with a CE of 30 V monitoring the following product ions for C(-HETE)P (m/z 323.1): m/z 105.1 (Qual 1) and m/z 137.0 (Qual 2), for carbobenzoxy-C(-HETE)P, herein referred to as Cbz-C(-HETE)P, (m/z 457.1): m/z 261.1 (Qual 1) and m/z 105.0 (Qual 2) and PA-C(-HETE)P (m/z 379.1): m/z 116.1 (Qual 1), m/z 137.0 (Qual 2) and m/z 105.0 (Qual 3). The IS d_3_-Atr was monitored with the product ions at m/z 127.1 (Qual 1) and m/z 93.1 (Qual 2) using a CE of 42 V. For analysis in the PIS mode, product ions of the derivatized products were monitored under conditions described above in a mass range from m/z 50 to m/z 500. The entire μLC-ESI MS/MS system was controlled by the Analyst TF 1.7.1 software (ABSciex) and the Eksigent control software (version 4.2).

### Characterization of the μLC-ESI MS/MS (SRM) method for PA-C(-HETE)P

#### Selectivity

The selectivity of the μLC-ESI MS/MS (SRM) method was tested by analyzing prepared blank plasma derived from six individuals not exposed to SM but subjected to derivatization looking for any interference at the relevant retention time (t_R_) of PA-C(-HETE)P.

#### Stability in the autosampler at 15 °C

The stability of PA-C(-HETE)P in a prepared plasma reference (50 μM SM) stored in the autosampler at 15 °C was investigated by hourly analysis by μLC-ESI MS/MS (SRM) over a period of 24 h. Peak areas obtained from the XICs of Qual 1 as well as their corresponding peak area ratio to IS were used to follow the relative concentration-time profiles.

#### Linear range, limit of detection, and limit of identification

For elaboration of the linearity and to estimate the LOD and LOI, 12 plasma standards were produced by adding diluted solutions of SM in iPrOH to plasma yielding 12 different concentrations. SM solution (20 μL) was added to 980 μL plasma each (n = 3) resulting in concentrations of 50 μM, 20 μM, 4 μM, 800 nM, 400 nM, 160 nM, 80 nM, 32 nM, 16 nM, 6.4 nM, 3.2 nM, and 1.28 nM. Standards were incubated, prepared, derivatized with propionic anhydride, and analyzed by μLC-ESI MS/MS (SRM) following the standard protocol. Peak areas from the XICs of Qual 3 were plotted against the SM concentration for linear regression. The LOD was defined as the lowest concentration of SM still allowing the detection of PA-C(-HETE)P in all three replicates. The LOI was defined as the lowest concentration of SM, where the detection of PA-C(-HETE)P was possible in all three replicates still fitting the respective peak area ratios of the plasma reference (50 μM SM).

#### Determination of the derivatization yield of PA-C(-HETE)P and Cbz-C(-HETE)P

References derived from the incubation with 50 μM and 12.5 μM SM were prepared and proteolyzed in quadruplicate with pronase, derivatized either with propionic anhydride or BCF or they were not derivatized and analyzed afterwards following the standard protocol. Peak areas obtained from the XICs of the individual Qual 1 of C(-HETE)P, PA-C(-HETE)P, and Cbz-C(-HETE)P were determined. The derivatization yield (ω) was calculated from the ratio of the C(-HETE)P peak area (A) after derivatization (a.d.) and without derivatization (w.d.) according to Eq. 1

1 $$ \upomega\ \left[\%\right]=\left\{1-\mathrm{A}{\left[\mathrm{C}\left(-\mathrm{HETE}\right)\mathrm{P}\right]}_{\mathrm{a}.\mathrm{d}.}/\mathrm{A}{\left[\mathrm{C}\left(-\mathrm{HETE}\right)\mathrm{P}\right]}_{\mathrm{w}.\mathrm{d}.}\right\}\times 100 $$

As results obtained for both references (12.5 μM and 50 μM SM) were identical, the mean and standard deviation (SD) were calculated.

#### Determination of the response factors of PA-C(-HETE)P and Cbz-C(-HETE)P

Based on the same set of samples analyzed to determine ω, the methodical response factor (rf_meth_) was calculated as the ratio of the peak areas of Qual 1 of C(-HETE)P (w.d.) and peak areas of Qual 1 of the derivatized biomarker (a.d.) according to Eq. 2:

2 $$ {\mathrm{rf}}_{\mathrm{meth}}=\mathrm{A}{\left\{\mathrm{X}-\mathrm{C}\left(-\mathrm{HETE}\right)\mathrm{P}\right\}}_{\mathrm{a}.\mathrm{d}.}/\mathrm{A}{\left\{\mathrm{C}\left(-\mathrm{HETE}\right)\mathrm{P}\right\}}_{\mathrm{w}.\mathrm{d}.} $$

with X representing either Cbz or PA.

The molecular response factor, rf_mol_, was calculated according to Eq. 3:

3 $$ {\mathrm{rf}}_{\mathrm{mol}}=\mathrm{A}{\left\{\mathrm{X}-\mathrm{C}\left(-\mathrm{HETE}\right)\mathrm{P}\right\}}_{\mathrm{a}.\mathrm{d}.}/\left\{\mathrm{A}{\left[\mathrm{C}\left(-\mathrm{HETE}\right)\mathrm{P}\right]}_{\mathrm{w}.\mathrm{d}.}-\mathrm{A}{\left[\mathrm{C}\left(-\mathrm{HETE}\right)\mathrm{P}\right]}_{\mathrm{a}.\mathrm{d}}\right\} $$

with X representing either Cbz or PA.

As results obtained from both references (12.5 μM and 50 μM SM) were identical, the mean and standard deviations (SD) were calculated for rf_meth_ and rf_mol_.

### Application of the novel procedure to real case plasma samples

In 2015, seven individuals were supposed to be exposed to SM in the Middle East after a mortar attack [4]. Plasma samples were taken 15 days after the attack. At that time, four of the seven persons still suffered from skin lesions and respiratory problems. The other three persons did not show any symptoms neither a few hours after the attack nor 15 days after that [4]. Plasma samples obtained from the seven persons were prepared following the herein described standard protocol for derivatization with propionic anhydride and analyzed for the presence of PA-C(-HETE)P. Medically examined patients consented to subsequent blood sample analysis. Samples were made available in 2015 in an anonymous mode not allowing the investigator (Bundeswehr Institute of Pharmacology and Toxicology) the retrieval of personal identifying data of the donors.

## Results and discussion

In general, the development of novel methods, the improvement of established procedures and the discovery of additional biomarkers are important efforts to extend the toolbox of methods for biomedical verification of SM exposure. The derivatization of biomarkers to generate novel diagnostic chemicals represents one of the relevant possibilities.

In the past decades, the derivatization of small molecules was often used to make, e.g., polar analytes more hydrophobic and thus suitable for GC analysis. Multiple reagents and methods were introduced as summarized by, e.g., Blau and Halket [21]. In contrast, methods to derivatize analytes for subsequent LC-MS analysis are less frequent. Several methods for the derivatization of proteins were introduced in the field of proteomics that allow the quantification of differently expressed proteins. Derivatization was done by, e.g., isotope labelling or metal coding [22–26]. In contrast, the derivatization of peptide biomarkers of CWA exposure is quite unusual. Merely, Chen et al. presented a procedure to convert C(-HETE)P with BCF [19]. However, the non-quantitative introduction of the hydrophobic carbobenzoxy moiety at the amine function of the N-terminus led to less favorable mass spectrometric and chromatographic properties. Therefore, an alternative derivatization procedure for a more favorable mass spectrometric behavior as well as a slighter increase of the hydrophobicity of the analyte appears as more beneficial.

Recently, van Faassen et al. reported on the propionylation of small molecules like tryptophan, serotonin, and 5-hydroxytryptophan at the amine as well as at the hydroxyl groups under mildest conditions within 15 min, using propionic anhydride [27]. In general, the derivatization with propionic anhydride was also shown to be applicable to nucleotides, phenolic compounds, and cytokines, thus improving chromatographic separation and MS/MS responses [28–30]. Even though van Faassen et al. did not present an application for peptide derivatization, we transferred this rapid and simple procedure to the C(-HETE)P biomarker.

### Derivatization of C(-HETE)P with propionic anhydride

**Fig. 1:**
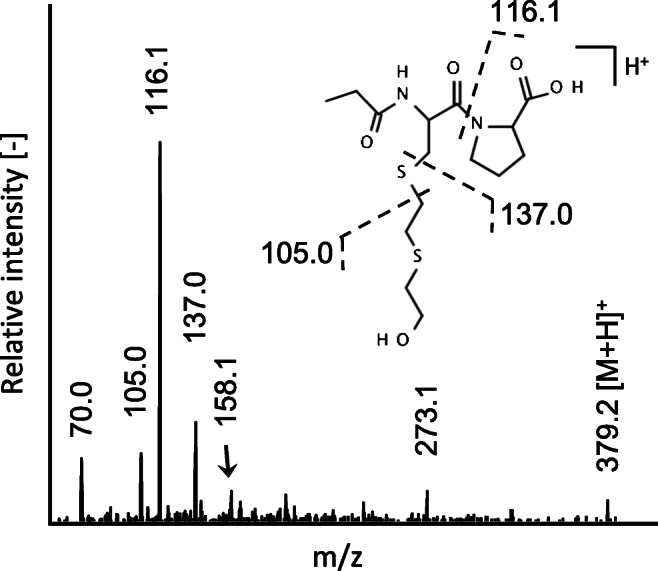
Product ion spectrum of single protonated PA-C(-HETE)P. The single protonated propionylated (PA) biomarker was subjected to collision-induced dissociation ([M + H]^+^, m/z 379.1) to extract the MS/MS data from a μLC-ESI MS/MS (PIS) run. Cleavage sites resulting in product ions used for the μLC-ESI MS/MS (SRM) method are depicted

Based on the results published by van Faasen et al. [27], the derivatization of the N-terminus as well as of the hydroxyl group of the HETE moiety of C(-HETE)P was assumed. Accordingly, μLC-ESI MS/MS in the PIS mode was performed after derivatization to monitor product ions of the single protonated adduct C(-HETE)P (m/z 323.1) as well as of the single derivatized product potentially present as PA-C(-HETE)P or C(-HETE-PA)P (m/z 379.1) and of the twofold derivatized biomarker PA-C(-HETE-PA)P (m/z 436.2). After the addition of 10 μL propionic anhydride, the XIC of Qual 1 of C(-HETE)P showed no peak anymore (data not shown), thus proving quantitative derivatization of the dipeptide. In addition, only one peak of a single derivatized product was observed at t_R_ 9.7 min, thus demonstrating the selective derivatization at only one functional group of C(-HETE)P. The corresponding product ion spectrum extracted from the μLC-ESI MS/MS PIS run is shown in Fig. 1.

**Fig. 2:**
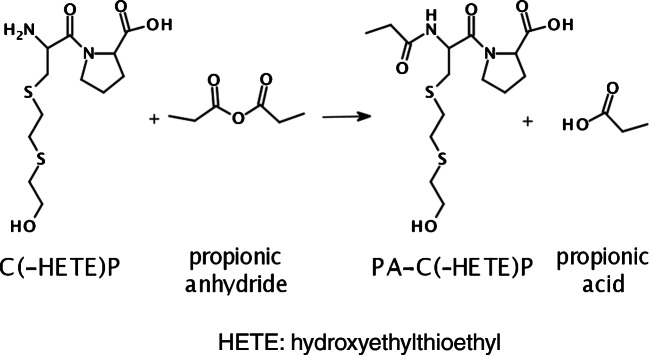
Derivatization procedure of C(-HETE)P with propionic anhydride

The product ion at m/z 116.1 belonged to the proline residue and the ion at m/z 70.0 represented the immonium ion of proline. The presence of the product ions at m/z 105.0 (HETE moiety) and at m/z 137.0 (HETE moiety plus sulfur atom of the C^34^ residue) strongly supported that the derivatization happened at the N-terminus of the C^34^ residue and not at the hydroxyl function of the HETE moiety as illustrated in Fig. 2. The latter derivative might have resulted in product ions at m/z 161.1 (PA-HETE moiety) and at m/z 193.1 (PA-HETE plus sulfur atom of C^34^). None of these ions was detected.

In addition, no peak was found for a potentially twofold derivatized biomarker, thus underlining the selectivity of the derivatization at the N-terminus. The same exclusive selectivity was found even though applying a larger molar excess of propionic anhydride (125 μL corresponding to a molar excess of 1.5 × 10^6^) in the presence of triethylamine (TEA) as a strong base. These findings are in agreement with other studies showing that the propionylation of primary aliphatic hydroxyl groups often requires either large bases like imidazole or non-aqueous solvents or propionic anhydride as the solvent itself [30, 31]. Hence, the hydroxyl group of the HETE moiety was obviously not reactive under the conditions applied.

**Fig. 3:**
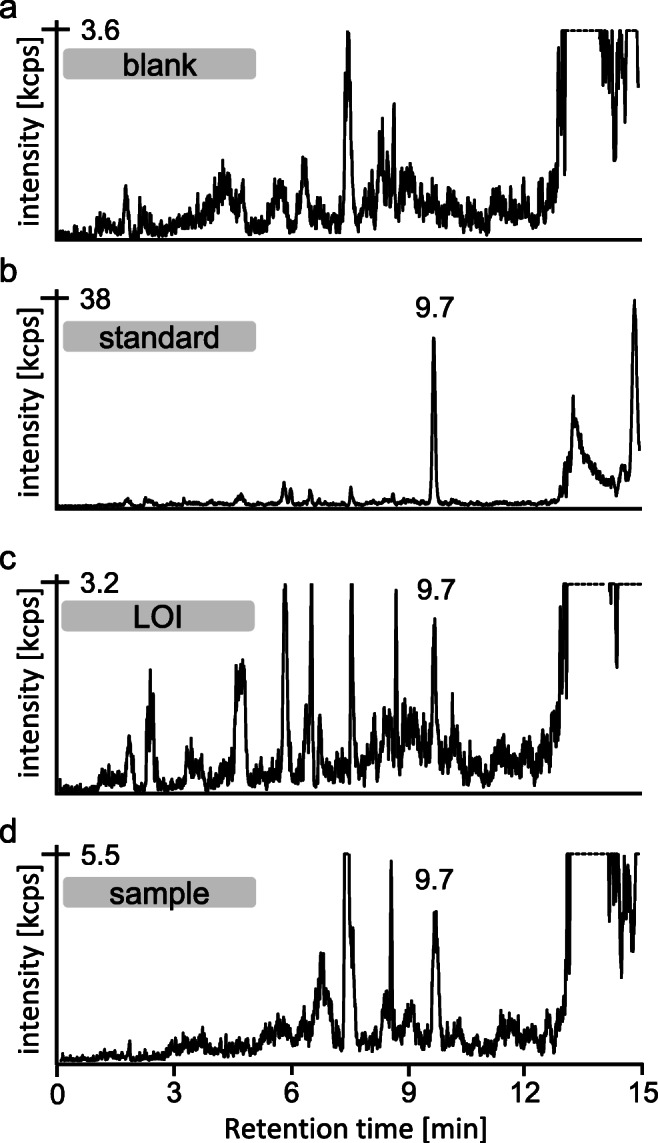
Extracted ion chromatograms of the derivatized biomarker of SM exposure PA-C(-HETE)P in human plasma. **a** Blank plasma not exposed to SM, **b** standard produced after plasma incubation with 4 μM SM, **c** plasma incubated with SM (160 nM) corresponding to the lower limit of identification (LOI), and **d** sample of a patient poisoned with SM in the Middle East [4]. The samples were prepared by pronase cleavage of adducted HSA in plasma and subsequent derivatization with propionic anhydride. Analyses were performed by μLC-ESI MS/MS (SRM). For reasons of clarity, only the product ion trace of m/z 105.0 is shown

The structural assignment of product ions was confirmed by MS/HRMS coupled to μLC as summarized in Table 1. The product ion at m/z 116.071, corresponding to the y_1_ fragment of the peptide backbone, was the most abundant product ion. This product ion was also observed after CID of C(-HETE)P as described by John et al. [16], but it was found at much lower relative intensity. The favored dissociation site at the proline amide bond of the PA derivative may be explained by the preferred site of protonation during the ESI process. In the non-derivatized C(-HETE)P molecule, the free amine group is the most favorable site of protonation. The protonated N-terminus enhances the energy, thus favoring the cleavage of the positively charged HETE moiety (m/z 105.037, Table 1). In contrast, the propionylated N-terminus of PA-C(-HETE)P is less favorable for protonation due to its lower basicity. Therefore, the tertiary amine group of the proline residue might be protonated primarily, thus resulting in the cleavage of the amide bond of proline as the predominant fragmentation site. In addition, the product ions at m/z 137.009 and at m/z 105.0373, which are characteristic for the HETE moiety, were also detected (Table 1). Accordingly, product ions at m/z 116.1 (Qual 1), m/z 137.0 (Qual 2), and m/z 105.0 (Qual 3) were used to set up the μLC-ESI MS/MS (SRM) method. Exemplarily, the XIC of Qual 3 of a standard (4 μM SM) is shown in Fig. 3b.

**Table 1:**
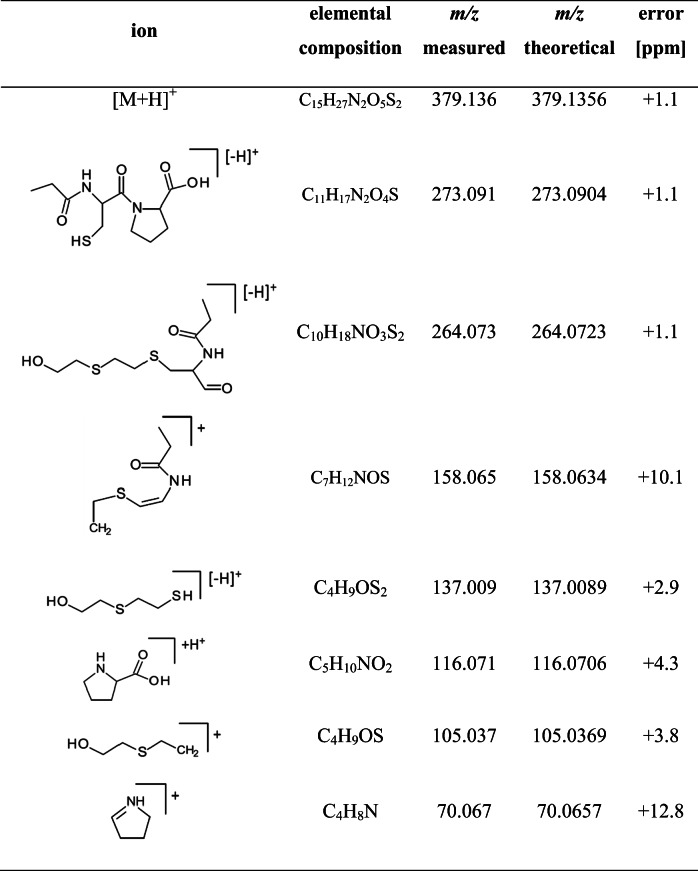
Product ions of single protonated PA-C(-HETE)P

Data was extracted from a μLC-ESI MS/HRMS run of the HETE-HSA adduct after enzymatic cleavage with pronase and subsequent propionylation (PA) monitoring product ions of m/z 379.1. This reference was produced by incubation of plasma with SM (50 μM). The structures represent one possible isomer each

### Optimization of derivatization conditions

The time for derivatization was varied by stopping the reaction with added water after various periods causing the hydrolysis of remaining propionic anhydride to propionic acid. It was found that quantitative conversion of C(-HETE)P has already happened after 10 s after the addition of 10 μL propionic anhydride. Lower volumes of the reagent did not allow quantitative conversion despite prolonged reaction times. Accordingly, the standard protocol made use of 10 μL propionic anhydride reacting for 10 s at RT.

### Derivatization of C(-HETE)P with BCF

C(-HETE)P was derivatized with BCF according to the published protocol of Chen et al. yielding Cbz-C(-HETE)P [19]. Accordingly, this derivative was monitored by the product ions at m/z 261.1 (Qual 1) and m/z 105.0 (Qual 2) in μLC-ESI MS/MS (SRM) analysis (t_R_ 13.2 min). In our study, the yield was found to be 92.8%. Surprisingly, this yield was significantly higher than that originally reported by Chen et al. (47%) [19].

#### Determination of the derivatization yield

The derivatization of C(-HETE)P with propionic anhydride was quantitative and therefore slightly better than the reaction with BCF. For both reagents, calculations of ω showed identical results independent of the concentrations of C(-HETE)P that were derived from references (50 μM and 12.5 μM SM). Accordingly, the mean of ω for PA-C(-HETE)P was 100% ± 0% and that of Cbz-C(-HETE)P 92.8% ± 2.0% (Table 2).

**Table 2:** Methodical and molecular response factors and yield of PA-C(-HETE)P and Cbz-C(-HETE)P

Analyte	ω [%]	rf_meth_ [−]	rf_mol_ [−]
Cbz-C(-HETE)P	92.8 ± 2.0	0.15 ± 0.03	0.16 ± 0.03
PA-C(-HETE)P	100 ± 0	0.37 ± 0.07	0.37 ± 0.07

Data characterize the derivatization of C(-HETE)P with propionic anhydride yielding PA-C(-HETE)P and with benzyl chloroformate yielding Cbz-C(-HETE)P

The rf values represent the mean and standard deviation of results obtained from plasma references incubated with 50 μM and 12.5 μM SM (n = 4, each). Values were calculated according to Eqs. 1–3

*ω* yield of derivatization, *rf*_*meth*_ methodical response factor, *rf*_*mol*_ molecular response factor, *Cbz* carbobenzoxy, *PA* propionyl

### Characterization of the μLC-ESI MS/MS (SRM) procedure for PA-C(-HETE)P

#### Selectivity

Following the standard protocol of sample preparation including two steps of protein precipitation, the reliable detection of PA-C(-HETE)P was possible without any interferences at t_R_ 9.7 min. Exemplarily, the XIC of the ion at m/z 105.0 (Qual 3) is illustrated in Fig. 3a.

#### Stability in the autosampler at 15 °C

The peak areas of the XIC of Qual 1 of PA-C(-HETE)P were found to be constant (relative standard deviation, RSD: 2%) over the entire test period of 24 h. Peak area ratios of PA-C(-HETE)P to d_3_-Atr used as IS remained constant as well (RSD: 3%) thus documenting no time-dependent decomposition of the derivative. Furthermore, t_R_ of PA-C(-HETE)P remained constant at 9.7 min ± 0.1 min not showing any shift, thus documenting robust and reliable chromatography. The high stability is highly beneficial when analyzing larger sets of samples.

#### Linearity, limit of detection, and limit of identification

The peak areas of PA-C(-HETE)P showed excellent linear relationship to the concentration of SM in plasma in a range from 32 nM to 50 μM (Qual 3), thus covering concentrations of toxicological relevance. The linearity documented the reproducibility of the derivatization procedure and its applicability to a broad concentration range of C(-HETE)P. The LOD for PA-C(-HETE)P was determined to be 32 nM.

Based on quadruplicate analysis of references, ion ratios (peak area ratios) of the different product ions were calculated yielding 26.5% ± 0.2% for Qual 2/Qual 1 and 18.6% ± 0.1% for Qual 3/Qual 1. At least one of these ratios should be met by any sample or standard defining the LOI [32]. Accordingly, the LOI for PA-C(-HETE)P was found at 160 nM based on the ratio of Qual 3/Qual 1. The corresponding XIC of Qual 3 is shown in Fig. 3c.

### Determination of rf_meth_ and rf_mol_

The relevant measures to characterize a novel biomarker for forensic trace analysis are usually the LOD and LOI. Accordingly, we determined both values for PA-C(HETE)P. However, these values are highly dependent on multiple factors like matrices, the sample preparation, working and dilution steps, and the mass spectrometer used. Therefore, the comparison of LOD and LOI is not the only way to evaluate the suitability of biomarkers. We herein introduce the rf_meth_ and rf_mol_ values which address a relative mass spectrometric response as an independent measure. The rf_meth_ value characterizes the relative signal yield (peak area) of the derivatized biomarker compared to the signal yield (peak area) of the non-derivatized biomarker. This value provides a measure of the procedure-dependent signal yield. In contrast, the rf_mol_ value characterizes the relative signal yield (peak area) of the derivatized molecule considering the derivatization yield.

For PA-C(-HETE)P, rf_meth_ and rf_mol_ were both found to be 0.37, thus documenting a lower signal yield than obtained for non-derivatized C(-HETE)P (Table 2). This might be due to the modified N-terminus of PA-C(-HETE)P, which protonation is less favorable than that of the free amine group of C(-HETE)P, thus reducing the ionization efficacy. However, rf_meth_ and rf_mol_ of PA-C(-HETE)P were more than two times higher than those of Cbz-C(-HETE)P found to be as low as about 0.16 (Table 2). Therefore, the propionylated derivative exhibits reasonably improved mass spectrometric properties when compared to the Cbz derivative. Even though the mass spectrometric response is lower than that of the non-derivatized biomarker, PA-C(-HETE)P might be valuable with respect to its different t_R_ if donor-specific plasma-derived interferences deteriorate C(-HETE)P analysis.

### Application of the novel procedure to real case samples

The μLC-ESI MS/MS (SRM) method introduced herein was applied to plasma samples obtained from victims of SM poisoning. As reported quite recently by our group, the SM exposure of four out of seven persons was proven by the detection of diverse HSA-derived HETE adducts including, e.g., C(-HETE)P and C(-HETE)PF [4]. The same set of samples was analyzed in the present study to monitor PA-C(-HETE)P. In accordance with our earlier report, poisoning by SM was detected only in the plasma of the four persons who also showed clinical signs and symptoms. As an example, the chromatogram of one sample is shown in Fig. 3d. The congruency of our novel and earlier data document that the procedure for derivatization with propionic anhydride is applicable to plasma samples from real exposure scenarios and provides reliable results. Even though sample draw in the Middle East was done 15 days after exposure [4], the remaining HSA adduct concentrations were sufficient to be traceable by the PA-C(-HETE)P biomarker.

## Conclusions

We herein present a plasma sample preparation procedure and bioanalytical μLC-ESI MS/MS (SRM) method for the precolumn derivatization of the dipeptide C(-HETE)P and the selective and sensitive detection of the derivative. The procedure was proven to be suitable for analysis of samples from real cases of SM poisoning. A simple split of the prepared sample that is ready for the detection of C(-HETE)P, and its subjection to the rapid and quantitative derivatization, may enable economic and fast monitoring of both markers without the need for an additional proteolytic step as it would be needed for C(-HETE)PF. The t_R_ of PA-C(-HETE)P (9.7 min) is significantly different from that of C(-HETE)P (t_R_ 4.6 min) and can therefore provide a valuable alternative if any sample shows matrix-derived interferences hindering C(-HETE)P monitoring. Therefore, we suggest this derivative as a novel biomarker in addition to the already internationally accepted non-derivatized peptide C(-HETE)P.

## Abbreviations

A: Peak area
ACN: Acetonitrile
a.d.: After derivatization
BCF: Benzyl chloroformate
Cbz: Carbobenzoxy moiety
CE: Collision energy
CID: Collision-induced dissociation
CUR: Curtain gas
CWA: Chemical warfare agent
CWC: Chemical Weapons Convention
CXP: Cell exit potential
d_3_-Atr: Three-fold deuterated atropine
DP: Declustering potential
EDTA: Ethylenediaminetetraacetic acid
EP: Entrance potential
ESI: Electrospray ionization
FA: Formic acid
GS1: Source gas 1
GS2: Source gas 2
HETE: Hydroxyethylthioethyl moiety
HSA: Human serum albumin
iPrOH: Isopropanol
IS: Internal standard
LOD: Limit of detection
LOI: Limit of identification
μLC: Microliquid chromatography
MS/MS: Tandem-mass spectrometry
MS/HRMS: High-resolution tandem-mass spectrometry
NMR: Nuclear magnetic resonance
OPCW: Organisation for the Prohibition of Chemical Weapons
PA: Propionyl moiety
PBS: Phosphate-buffered saline
PIS: Product ion scan
Qual: Qualifying ion
RT: Room temperature
SM: Sulfur mustard
SRM: Selected reaction monitoring
TDG: Thiodiglycol
TEA: Triethylamine
TFA: Trifluoroacetic acid
TIC: Total ion chromatogram
t_R_: Retention time
w.d.: Without derivatization
ω: Derivatization yield
XIC: Extracted ion chromatogram
